# The efficacy and acceptability of pharmacological monotherapies and e-cigarette on smoking cessation: a systemic review and network meta-analysis

**DOI:** 10.3389/fpubh.2024.1361186

**Published:** 2024-05-22

**Authors:** Yajing Meng, Sike Xiang, Lang Qu, Ying Li

**Affiliations:** ^1^Mental Health Center, West China Hospital of Sichuan University, Chengdu, China; ^2^Department of Medicine, Sinai Hospital of Baltimore, Baltimore, MD, United States; ^3^Department of Cardiology, West China School of Public Health and West China Fourth Hospital, Sichuan University, Chengdu, China

**Keywords:** smoking cessation, e-cigarette, nicotine replacement therapy, cytisine, varenicline, bupropion

## Abstract

**Background and aims:**

Several pharmacological interventions, such as nicotine replacement therapy (NRT), varenicline, and bupropion, have been approved for clinical use of smoking cessation. E-cigarettes (EC) are increasingly explored by many RCTs for their potentiality in smoking cessation. In addition, some RCTs are attempting to explore new drugs for smoking cessation, such as cytisine. This network meta-analysis (NMA) aims to investigate how these drugs and e-cigarettes compare regarding their efficacy and acceptability.

**Materials and methods:**

This systematic review and NMA searched all clinical studies on smoking cessation using pharmacological monotherapies or e-cigarettes published from January 2011 to May 2022 using MEDLINE, COCHRANE Library, and PsychINFO databases. NRTs were divided into transdermal (TDN) and oronasal nicotine (ONN) by administrative routes, thus 7 network nodes were set up for direct and indirect comparison. Two different indicators measured the efficacy: prevalent and continuous smoking abstinence. The drop-out rates measured the acceptability.

**Results:**

The final 40 clinical studies included in this study comprised 77 study cohorts and 25,889 participants. Varenicline is more effective intervention to assist in smoking cessation during 16–32 weeks follow-up, and is very likely to prompt dropout. Cytisine shows more effectiveness in continuous smoking cessation but may also lead to dropout. E-cigarettes and oronasal nicotine are more effective than no treatment in encouraging prevalent abstinence, but least likely to prompt dropout. Finally, transdermal nicotine delivery is more effective than no treatment in continuous abstinence, with neither significant effect on prevalent abstinence nor dropout rate.

**Conclusion:**

This review suggested and agreed that Varenicline, Cytisine and transdermal nicotine delivery, as smoking cessation intervention, have advantages and disadvantages. However, we had to have reservations about e-cigarettes as a way to quit smoking in adolescents.

## 1 Introduction

Unquestionably, tobacco smoking is one of the modifiable factors that heavily contribute to the global health burden. According to a global burden of disease study, there will be an increasing number of 7.69 million deaths and 200 million disability-adjusted life-years attributable to tobacco smoking within this decade if interventions are abscent^1.^ Multiple behavioral and pharmacologic interventions, both in combination and individually, were proved effective and applied in practice (1). Even though previous randomized controlled trials (RCTs) and meta-analyses showed evidence supporting the effectiveness of behavioral interventions in smoking cessation (1, 2), their effectiveness is relatively modest compared with approved pharmacological interventions (1).

Current to the date when this study was performed, there were 7 pharmacological interventions widely approved by most countries: nicotine replacement therapy (NRT, including nicotine mouth spray, inhaler, gum, patch, and lozenge), varenicline, and bupropion. However, inconsistent effectiveness reported by RCTs and meta-analysis of the above pharmacological therapies is not excellent enough, and the relapse rate remains high (3). Besides, the relatively high cost of NRT and varenicline also prevent patients who are in low-income classes from approaching such smoking cessation aids (4). It is still important to innovate novel pharmacological interventions for more cost-effective and acceptable aids in assisting smoking cessation.

As a new product with the potential in assisting smoking cessation, the e-cigarette has already shown evidence of effectiveness and non-inferiority to NRTs in assisting smoking cessation from the previous meta-analysis of both RCTs and observational studies (5, 6). Cytisine, due to its similar mechanism with varenicline as a selective partial agonist of nicotinic acetylcholine receptors and low cost of production, has also been previously investigated and proved to be effective and globally affordable in assisting smoking cessation (4, 7).

This network meta-analysis aims to systemically and quantitatively evaluate and compare the overall effectiveness and acceptance of all above-mentioned interventions.

## 2 Method

### 2.1 Search strategies and literature resources

We searched MEDLINE, COCHRANE Library & PsychINFO for RCTs reporting pharmacological monotherapies and/or e-cigarettes (and equivalents) on smoking cessation. Due to the purpose of comparability and consistency of study cohorts, the time of publication was restricted to be from 2011 Jan 1st [in which the first RCT reporting e-cigarette was released (5)] to 2022 May 31st (in which this network meta-analysis was firstly proposed) during searching. Additionally, references to already-published reviews and meta-analyses with a similar topic were also screened for consideration of inclusion.

### 2.2 Eligibility criteria and study selection

Eligibility criteria were proposed before we perform this network meta-analysis. Inclusion of studies was considered if the study met the following: (1) RCTs; (2) reported in English; (3) study cohorts were recruited in a community-based setting; (4) study cohorts had a persistent smoking history; (5) pharmacological monotherapies or e-cigarette (or its equivalent) were used as an intervention in ≥1 study cohort. Furtherly, studies were excluded if: (1) duplicate records; (2) the study cohort was with a major health condition (e.g., cancer, chronic respiratory diseases, heart and vascular diseases, and schizophrenia or bipolar disorder); (3) follow-up of study endpoint was less than 4 weeks; (4) study outcomes (smoking abstinence, prevalent and/or continuous) were not supported by objective evidence (e.g., saliva cotinine, exhaled CO, serum cotinine, urine cotinine).

Two reviewers (L. Qu and S. Xiang) independently searched and selected studies according to the above strategies and criteria, with disagreement resolved by discussion. All citations retrieved from the database were firstly screened for eligibility at Title/Abstract level, and identified studies were furtherly acquired and examined in full text. Forty studies were eligible and included in the final analysis (8–47).

### 2.3 Data identification and extraction

We identified three study outcomes for this meta-analysis due to our study interest, which are defined and listed as the following: (1) ***Prevalent smoking abstinence (PSA)**:* the percentages of the population who currently quit or reduced cigarette use during the follow-up investigation in between 16 and 32 weeks; (2) ***Continuous smoking abstinence (CSA)***: the percentages of the population who consistently maintain smoke quitting or reduction from the first to the last follow up the investigation; (3) ***Treatment drop-out rates (TDR)***: the percentages of the population who dropped out from the study or lost to follow-up during the treatment period.

Data identification and extraction were performed by 2 reviewers (L. Qu and S. Xiang) independently. Additional to direct data and indirect data used for the calculation of study outcomes, the baseline characteristics of each study were evaluated and extracted: sampling population, age, location, sex, recruitment setting, smoking history, comparisons, pharmacological dosage, duration of exposure, length of follow up, and lab methods measuring smoking abstinence (see as Table 1).

**Table 1:** Baseline characteristics of each identified studies.

Study	Female, total		Location	Smoking history		Placebo	Study groups	Baseline intervention?	Method used for verification	Duration of intervention	End-points	Statistical procedures	The critical summary of the intervention
				Frequency (cpd)	Duration (year)								
Caponnetto, 2011	40	120	Italy	>20	N/A	No	ONN vs. CTL (nicotine inhaler)	Yes	Exhaled CO	4 weeks	4 and 24 weeks	A logistic regression model	A high Glover-Nilsson Smoking Behavioural Questionnaire score is a strong independent predictor for successful quitting at 24 weeks in the intervention group.
Cox, 2011	357	540	USA	>10	N/A	Yes	BUP vs. CTL	No	Exhaled CO	7 weeks	26 weeks	A logistic regression model	No statistically significant difference in long-term smoking abstinence rates at week 26 was observed between sustained release bupropion and placebo groups. Cotinine-verified smoking abstinence rate at end of medication week 7 was higher in the sustained release bupropion vs placebo group.
West, 2011	24	2472	Bangladesh, Parkistan	Daily	N/A	Yes	CYT vs. CTL	No	Exhaled CO	25 days	6 and 12 months	Logistic regression	The rate of sustained 12-month abstinence was 8.4% in the cytisine group as compared with 2.4% in the placebo group. The 7-day point prevalence for abstinence at the 12-month follow-up was 13.2% in the cytisine group versus 7.3% in the placebo group.
Tønnesen, 2012	210	479	Denmark	Daily	N/A	Yes	ONN vs. CTL (nicotine mouth spray)	No	Exhaled CO	24 weeks	2, 3, 4, 5, 6, 24, and 52 weeks	Pearson's Chi-squared test and The Mann–Whitney U-test	Nicotine mouth spray delivered significantly higher 6-, 24- and 52-week continuous abstinence rates than placebo.
Dios, 2012	17	32	USA	≤10	>3 months	Yes	TDN vs. CTL	No	Exhaled CO, salivary cotinine	12 weeks	3,4 and 6 months	Fischer’s exact p-values, the nonparametric Kruskal-Wallis test and Graphical methods	There were no abstinent participants in the placebo and NRT groups. However, 30% of participants in the varenicline group were abstinent at the 3-, 4-, and 6-month follow-up.
Heydari, 2012	112	272	Iran	N/A	N/A	No	TDN vs. VAR vs. CTL	Yes	Exhaled CO	8 weeks	1 and 12 months	The Kruskall Wallis and analysis of variance (ANOVA) tests	Varenicline treatment was slightly more effective than but not significantly different from NRT.
Wong, 2012	135	286	Canada	>10	N/A	Yes	VAR vs. CTL	No	Exhaled CO, urine cotinine	12 weeks	3, 6 and 12 months	Multivariable logistic regression	A perioperative smoking cessation intervention with varenicline increased abstinence from smoking 3, 6, and 12 months after elective noncardiac surgery with no increase in serious adverse events.
Cinciripini, 2013	114	294	USA	>10	N/A	Yes	VAR vs. BUP vs. CTL	Yes	Exhaled CO	12 weeks	3, 4 and 6 months	Mixed model regression	Varenicline exerts a robust and favorable impact on smoking cessation relative to placebo and may have a favorable on symptoms of depression and other affective measures in a community sample
Bullen2, 013	405	657	New zealand	>10	N/A	Yes	EC vs. TDN vs. CTL	Yes	Exhaled CO	12 weeks	1, 3 and 6 months	Multivariate regression, Kaplan-Meier curves and the log rank test	E-cigarettes, with or without nicotine, were modestly eff ective at helping smokers to quit, with similar achievement of abstinence as with nicotine patches, and few adverse events.
Caponnetto, 2013	76	200	Italy	>10	N/A	Yes	EC vs. CTL	No	Exhaled CO	6 or 12 weeks	2, 4, 6, 8, 10, 12, 24 and 52 weeks	Kolmogorov-Smirnov Test	In smokers not intending to quit, the use of e-cigarettes decreased cigarette consumption and elicited enduring tobacco abstinence without causing significant side effects.
Ward, 2013	58	269	Syria	>10	>1	Yes	TDN vs. CTL	Yes	Exhaled CO	6 weeks	6 and 12 months	Generalized estimating equation	Treatment adherence was excellent and nicotine patch produced expected reductions in urges to smoke and withdrawal symptoms, but no treatment effect was observed.
Gonzales, 2014	249	498	Global, multicenter	>10	N/A	Yes	VAR vs. CTL	No	Exhaled CO	12 weeks	13, 16, 24, 32, 40, 48, and 52 weeks	A logistic regression model	Varenicline is efficacious and well tolerated in smokers who have previously taken it. Abstinence rates are comparable with rates reported for varenicline-naive smokers.
Cooper, 2014	1050	1050	UK	>10	N/A	Yes	TDN vs. CTL	Yes	Exhaled CO	8 weeks	2 years	A cost-effectiveness analysis	NRT patches had no enduring, significant effect on smoking in pregnancy; however, 2-year-olds born to women who used NRT were more likely to have survived without any developmental impairment.
Scherphof, 2014	136	257	Netherlands	>7	N/A	Yes	TDN vs. CTL	No	Salivary cotinine	6 or 9 weeks	6 and 12 months	N/A	NRT fails in helping adolescents quit smoking at 6 and 12 months follow-ups.
Berlin, 2014	402	402	France	>5	N/A	Yes	TDN vs. CTL	No	Salivary cotinine	12 weeks	more than 20-28 weeks	A mixed effect logistic model	The nicotine patch did not increase either smoking cessation rates or birth weights despite adjustment of nicotine dose to match levels attained when smoking, and higher than usual doses.
Ebbert, 2015	659	1510	Global, multicenter	>10	N/A	Yes	VAR vs. CTL	No	Exhaled CO	24 weeks	28 weeks	A logistic regression model	Among cigarette smokers not willing or able to quit within the next month but willing to reduce cigarette consumption and make a quit attempt at 3 months, use of varenicline for 24 weeks compared with placebo significantly increased smoking cessation rates at the end of treatment, and also at 1 year.
Hsueh, 2015	66	463	Taiwan	>10	N/A	No	TDN vs. VAR	Yes	Exhaled CO	90 days	3 and 6 months	N/A	Varenicline users had a significantly higher abstinence rate than those using nicotine patch at 3-month and 6-month follow-up.
Gray, 2015	140	140	USA	>10	N/A	No	TDN vs. VAR	Yes	Exhaled CO	24 weeks	24 weeks and 1 years	A logistic regression model	In an exploratory four-week head-to-head trial in female smokers, varenicline, compared with nicotine patch, more than doubled the odds of end-of-treatment abstinence.
Tuisku, 2016	97	197	Finland	Daily	N/A	Yes	TDN vs. VAR vs. CTL	No	Salivary cotinine	8 or 12 weeks	52 weeks	N/A	Saliva cotinine verified abstinence at week 12 did not support self-reported abstinence. Varenicline may be more effective than the nicotine patch as a smoking cessation pharmacotherapy among young adult heavy smokers in the short-term.
Anthenelli, 2016	1985	3984	Global, multicenter	>10	N/A	Yes	TDN vs. VAR vs. BUP vs. CTL	Yes	Exhaled CO	12 weeks	9-12 weeks	Logistic regression	Varenicline was more effective than placebo, nicotine patch, and bupropion in helping smokers achieve abstinence, whereas bupropion and nicotine patch were more effective than placebo
Cunningham, 2016	511	999	Canada	>10	N/A	No	TDN vs. CTL	No	Salivary cotinine	5 wees	6 months	Separate logistic regression	The trial provides evidence of the effectiveness of mailed nicotine patches without behavioral support to promote tobacco cessation.
Baker, 2016	347	665	USA	>5	N/A	No	TDN vs. VAR	Yes	Exhaled CO	12 weeks	26 weeks	Linear regression model	Among adults motivated to quit smoking, 12 weeks of open-label treatment with nicotine patch, varenicline, or combination nicotine replacement produced no significant differences in confirmed rates of smoking abstinence at 26 weeks.
Tulloch, 2016	228	492	Canada	>10	N/A	No	TDN vs. VAR	Yes	Exhaled CO	22-24 weeks	5–52 weeks	An adjusted logistic regression model	Flexible and combination NRT and varenicline enhance success in the early phases of quitting. Varenicline improves abstinence in the medium term; however, there is no clear evidence that either varenicline or flexible, dual-form NRT increase quit rates in the long-term when compared to NRT monotherapy.
Ebbert, 2016	56	93	USA	>5	N/A	Yes	VAR vs. CTL	No	Exhaled CO	12 weeks	3 and 6 months	Logistic regression	Varenicline was safe and effective for increasing long-term smoking abstinence rates in a population of predominantly White light cigarette smoker.
Benli, 2017	71	405	USA	N/A	N/A	No	VAR vs. BUP	Yes	Exhaled CO	3 months	1, 2, 3, 6 and 12 months	N/A	No significant difference was found between the success rates of varenicline and bupropion used in smoking cessation based on the last 7 days at the end of one year. Those who used the medications for 45 days or longer were more successful in smoking cessation.
Carpenter, 2017	41	68	USA	>5	N/A	Yes	EC vs. CTL	No	Urine cotinine	3 weeks	4 months	Generalized estimating equations	Cigarette smokers are willing to use electronic nicotine delivery systems with trends towards reduced cigarette smoking and positive changes in cessation-related behaviors.
Oxford, 2018	848	1792	UK	N/A	N/A	No	TDN vs. CTL	Yes	Exhaled CO	4 weeks	4 weeks, 6 and 12 months	Multivariable logistic regression	Evidence was insufficient to confidently show that nicotine preloading increases subsequent smoking abstinence.
Halpern, 2018	1012	2012	USA	N/A	N/A	No	EC vs. CTL	Yes	Urine cotinine	6 months	1, 3, and 6 months	Logistic regression	Financial incentives added to free cessation aids resulted in a higher rate of sustained smoking abstinence than free cessation aids alone. Among smokers who received usual care (information and motivational text messages), the addition of free cessation aids or e-cigarettes did not provide a benefit.
Lee, 2019	0	150	South Korea	>10	>3	No	EC vs. ONN (Nicotine gum)	Yes	Exhaled CO, urine cotinine	12 weeks	9,12 and 24 weeks	Multivariable logistic regression	The effect of e-cigarettes on smoking cessation was similar compared with that of nicotine gum, a well-documented NRT. And e-cigarettes were well tolerated by the study population.
Masiero, 2019	78	210	Italy	>10	N/A	Yes	EC vs. CTL	Yes	Exhaled CO	3 months	6 months	A Kruskal–Wallis test	The efficacy and safety of e-cigarettes in a short-term period. E-cigarettes use led to a higher cessation rate.
Gilbert, 2019	114	294	USA	>5	N/A	Yes	VAR vs. BUP vs. CTL	Yes	Exhaled CO	12 weeks	3 and 6 months	Logistic regression	Varenicline exerts a robust and favorable impact on smoking cessation relative to placebo and may have a favorable on symptoms of depression and other affective measures with no clear unfavorable impact on neuropsychiatric adverse events in a community sample.
Oncken, 2019	137	137	USA	>5	N/A	Yes	ONN vs. CTL (nicotine inhaler)	Yes	Exhaled CO	6 weeks	32 weeks	Linear regression and logistic regression	Although the nicotine inhaler group did not have a higher quit rate during pregnancy than the placebo group, the outcome of preterm delivery occurred less frequently in the nicotine group.
Nides, 2020	651	1198	USA	Daily	N/A	Yes	ONN vs. CTL (nicotine mouth spray)	No	Exhaled CO	26 weeks	1, 2, 4, 6, 8, 12, 16, 20, and 26 weeks	The Cochran–Mantel– Haenszel test	The nicotine mouth spray is an effective and safe smoking cessation option for smokers motivated to quit, even in a naturalistic setting and without behavioral support.
Xiao, 2020	5	239	China	Daily	>1	Yes	ONN vs. CTL (nicotine lozegen)	Yes	Exhaled CO	12 months	6, 24 and 52 weeks	The Cochran–Mantel– Haenszel test	The 4mg nicotine lozenge provided a directionally significant improvement in smoking cessation rates compared with placebo in Chinese adult smokers with high nicotine dependence for the primary endpoint. The 2mg nicotine lozenge provided higher, but nonsignificant, smoking cessation rates than placebo.
Shiffman, 2020	210	369	USA	Daily	>3	Yes	ONN vs. CTL (nicotine gum)	Yes	Exhaled CO, urine cotinine	8 weeks	6 months	Multi-level generalized linear mixed models	Nicotine gum (2 mg), used intermittently, did not improve cessation rates among ITS, including those demonstrating some degree of dependence.
Gray, 2020	63	157	USA	Daily	N/A	Yes	VAR vs. CTL	No	Urine cotinine	12 weeks	12, 24, and 52 weeks	Logistic regression model	This trial did not show an advantage in abstinence with varenicline compared with placebo among adolescent smokers. The rates of treatment-emergent adverse events were similar to those in previous trials of adult smokers, raising no new tolerability signals.
Eisenberg, 2020	121	255	Canada	>10	>3	Yes	EC vs. CTL	Yes	Exhaled CO	12 weeks	12, 24 and 52 weeks	Multiple logistic regression models	Nicotine e-cigarettes plus counseling vs counseling alone significantly increased point prevalence abstinence at 12 weeks. However, the difference was no longer significant at 24 weeks, and trial interpretation is limited by early termination and inconsistent findings for nicotine and nonnicotine e-cigarettes, suggesting further research is needed.
Walker, 2021	473	679	New Zealand	Daily	N/A	No	CYT vs. VAR	Yes	Exhaled CO	12 weeks	1, 3, 6 and 12 weeks	Kaplan–Meier curves, the log‐rank test and Cox proportional hazards regression	Cytisine was at least as effective as varenicline at supporting smoking abstinence in New Zealand indigenous Māori or whānau (extended‐family) of Māori, with significantly fewer adverse events.
Nides, 2021	56	101	USA	>10	N/A	Yes	CYT vs. CTL	Yes	Exhaled CO	25 days	5 and 8 weeks	Variance (ANOVA) model	Based on simpler dose scheduling, excellent tolerability, and best-continued abstinence rate, cytisinicline 3-mg TID was selected for future Phase 3 studies.
Courtney, 2021	742	1452	Australia	Daily	N/A	No	CYT vs. VAR	Yes	Exhaled CO	25 or 84 days	6 months	The bayesian analysis	The study findings failed to demonstrate noninferiority of cytisine compared with varenicline regarding smoking cessation.

cpd, Cigarettes per day; USA, United States of America; UK, United Kingdom; TDN, transdermal nicotine; ONN, oronasal nicotine; VAR, varenicline; BUP, bupropion; CYT, cytisine; CTL, controls; CO, carbon monoxide. NRT, Nicotine replacement therapy.

### 2.4 Data analysis

#### 2.4.1 Comparative arms

All considered interventions were classified into 6 arms for comparison: (1) Varenicline (VAR); (2) Bupropion (BUP); (3) Transdermal nicotine delivery (TND) (nicotine patch); (4) Oronasal nicotine delivery (ONN) (nicotine gum, nicotine nasal spray, nicotine inhaler, nicotine tablet/lozenge); (5) Cytisine (CYT); (6) Electronic cigarette (EC) (or its equivalents).

#### 2.4.2 Data analysis

All outcomes were dichotomous variables measured as n/N (%). The odds ratio (OR) of each outcome was pooled for network meta-analysis (NMA). The NMA used the Bayesian method for multiple-treatment to pool the OR, under the assumption: the heterogeneity is independent of the comparative arms being used (48). We calculated the Bayesian 95% confidence interval (which is known as credible interval) to estimate the range of the OR results (49). As proposed by the previous study (50), the statistical models for NMA were chosen based on a model comparison criterion called the Deviance Information Criterion (DIC), which is the sum of the posterior expectation of the overall residual deviance and the posterior mean of the parameter of interest (50, 51). We firstly applied both random and fixed models for each outcome and calculated the DIC of both models, then the model with a lower DIC is chosen if the difference of DIC in each model is considerable (>5), otherwise the fixed model is chosen if the between-study difference of DIC in each model is insignificant (<5). The absolute value of the between-study variance in the random effect model was assessed by *Tau^2^*; the heterogeneity of variation across studies was estimated through *I^2^* statistics. Additionally, we used the node-splitting method to evaluate the local consistency by separating direct evidence from indirect evidence (50, 52). To rank the interventions for each outcome, we estimated the posterior distribution of the ranking probability and their corresponding estimated surface under the cumulative ranking curve (SUCRA) (50, 52). The SUCRA is an estimated index to show the cumulative rank probabilities for each intervention and simplifies the entire information about treatment ranking into a single number.

As guided by the Cochrane Handbook for Systematic Reviews of Interventions, the internal validity and quality of this systemic review and NMA were evaluated through the aspects of randomization, blinding of intervention allocation and outcome assessment, and incomplete outcome data (51). All data synthesis and statistical analysis were performed in R with the *gemtc* package: https://github.com/gertvv/gemtc

## 3 Results

### 3.1 Baseline characteristics

**Figure 1:**
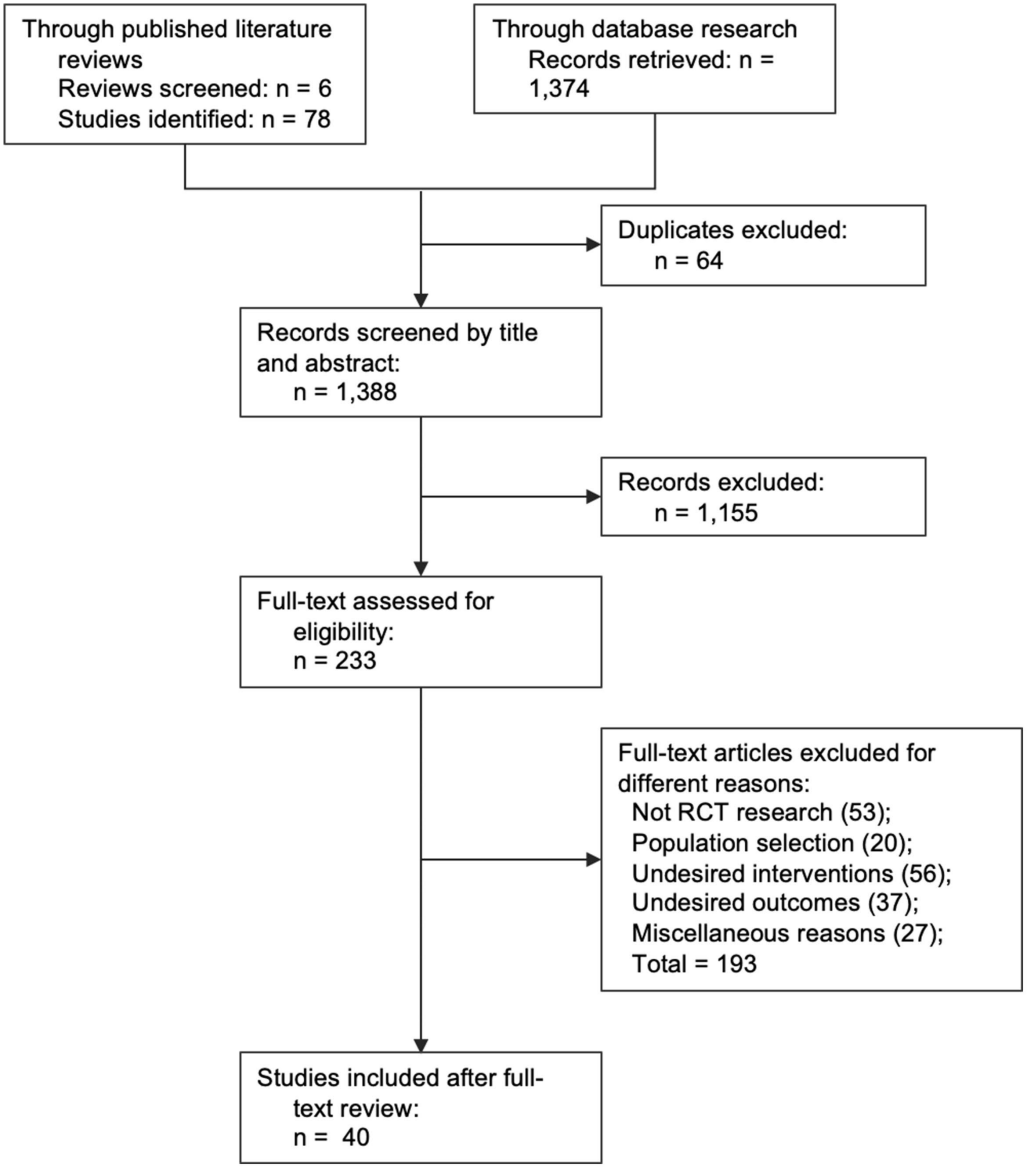
Flowchart of study inclusion.

The baseline characteristics of each study were summarized in Table 1. Forty studies included a total of 77 study cohorts and 25,889 participants, with an average age of 43.2 years old and 46.7% female (12,096) participants, and nearly half of the studies were performed in North America (19/40, 47.5%). Among the all 40 identified studies, three studies [Scherphof et al. (22), Berlin et al. (19), and Oncken et al. (39)] included pregnant patients only (18, 21, 38), seven studies (7/40, 17.5%) included more than 2 treatment arms for the NMA, 11 studies (11/40, 27.5%) had no placebo-controlled group, 15 studied (15/40, 37.5%) had no baseline intervention between each study’s comparative arms. For studies with multiple cohorts that used the same intervention with different dosages, only cohorts with higher nicotine dosage [7.2 mg nicotine EC cohort in Caponnetto et al. (17), 15 mg/16 h nicotine patches cohort in Tuisku et al. (30), 4 mg nicotine lozenge cohort in Xiao et al. (46), 3 mg cytisine three times per day cohort in Nides et al. (48)] were selected for the analysis (16, 29, 45, 47). Methods used for verification of smoking abstinence included CO concentration of exhaled air (29/40, 72.5%), salivary cotinine concentration (4/40, 10%), urine cotinine (3/40, 7.5%), and the combination of the above (4/40, 10%) (Figure 1).

### 3.2 Pooled effect

#### 3.2.1 Prevalent smoking abstinence (PSA)

**Figure 2:**
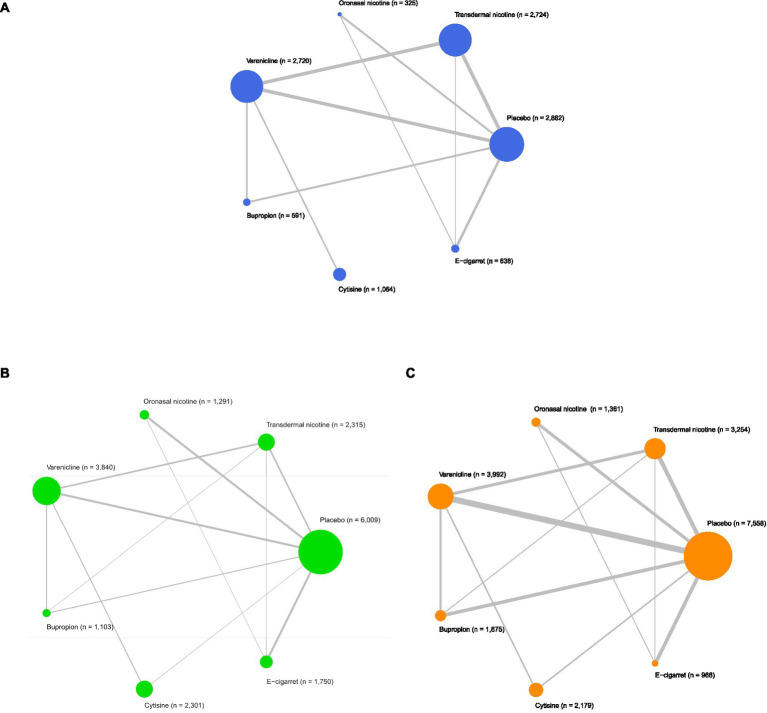
Network meta-analysis of eligible comparisons. **(A)** Network meta-analysis for comparisons of prevalent smoking abstinence (blue). **(B)** Network meta-analysis for comparisons of continuous smoking abstinence (green) and **(C)** Network meta-analysis for comparisons of treatment drop-out rates (orange). *n* indicates the number of total participants.

**Figure 3:**
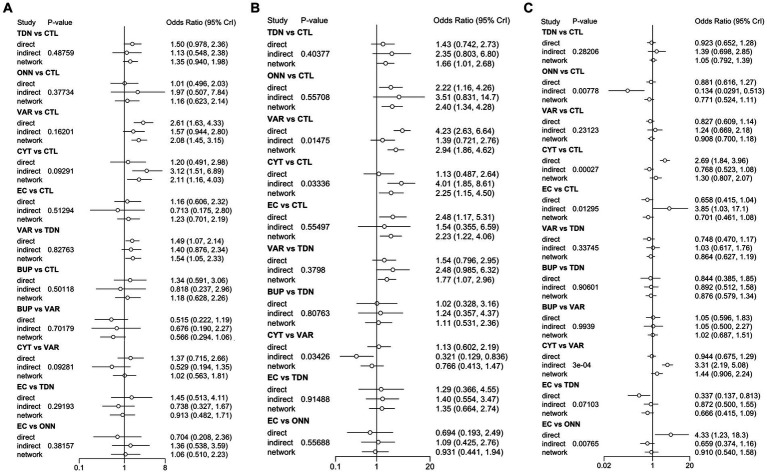
Inconsistency check between direct and indirect evidence in the network meta-analysis of *PSA* **(A)**, *CSA* **(B)**, and *TDR* **(C)**. PSA, prevalent smoking abstinence; CSA, continuous smoking abstinence; DOR, drop-out rate; TDN, transdermal nicotine; ONN, oronasal nicotine; VAR, varenicline; BUP, bupropion; CYT, cytisine; CTL, controls; 95% Crl, Credible interval.

Figures 2A, 3A described the network used for the main analyses of ***PSA***, comprising 60 study cohorts and 13,818 participants. We analyzed the pooled network effect of ***PSA*** for all interventions compared with the control group, using both random and fixed effect models initially. The random-effect model was selected for the final report due to significantly lower DIC (112.84 in the random model, 140.56 in the fixed model), indicating a better efficient result. As presented in Table 2, the confidential presentation of results in terms of mean OR with 95% credible intervals (Crl) compared with the control group was summarized. The pooled effects of all nicotine-containing products (***ONN***, ***TDN, EC***) as well as buspirone did not exhibit significant superiority over the control group in terms of prevalent smoking abstinence. The ***CYT*** and VAR, both demonstrating significant superiority, exhibited similar odds of prevalent smoking abstinence, approximately twice that of the control group. Despite ***CYT*** and VAR showing significant superiority over the control group compared to other intervention groups, these two interventions mostly did not exhibit a significantly different odds ratio for ***PSA*** relative to other active intervention groups. The only notable significance observed among active intervention groups was in ***VAR***, with approximately 50% higher odds compared to ***TDN*** (Table 2 and Figure 2 are shown here).

**Table 2:** Posterior distributions of odds ratios for random effect consistency model of each intervention and control group.

Prevalent smoking abstinence (A)							
Treatments	Odds ratio (95% credible intervals)						
	CTL	TDN	ONN	VAR	BUP	CYT	EC
CTL		1.35 (0.96, 1.93)	1.18 (0.64, 2.12)	2.09 (1.44, 3.24)	1.2 (0.62, 2.3)	2.1 (1.16, 3.97)	1.24 (0.73, 2.25)
TDN	0.74 (0.52, 1.04)		0.86 (0.43, 1.74)	1.55 (1.05, 2.34)	0.88 (0.43, 1.79)	1.55 (0.83, 3.01)	0.92 (0.49, 1.73)
ONN	0.85 (0.47, 1.57)	1.16 (0.57, 2.34)		1.8 (0.86, 3.77)	1.02 (0.43, 2.56)	1.81 (0.77, 4.48)	1.05 (0.51, 2.23)
VAR	0.48 (0.31, 0.69)	0.65 (0.43, 0.95)	0.56 (0.26, 1.16)		0.57 (0.3, 1.07)	1.01 (0.56, 1.76)	0.59 (0.3, 1.12)
BUP	0.83 (0.44, 1.6)	1.14 (0.56, 2.32)	0.98 (0.39, 2.3)	1.76 (0.94, 3.38)		1.77 (0.75, 4.03)	1.04 (0.46, 2.4)
CYT	0.48 (0.25, 0.86)	0.65 (0.33, 1.2)	0.55 (0.22, 1.3)	0.99 (0.57, 1.8)	0.57 (0.25, 1.33)		0.59 (0.26, 1.27)
EC	0.81 (0.45, 1.37)	1.09 (0.58, 2.06)	0.95 (0.45, 1.95)	1.69 (0.89, 3.34)	0.96 (0.42, 2.18)	1.71 (0.79, 3.92)	
DIC	112.84						
I^2^	6%						
Tau^2^	0.21						

Continuous smoking abstinence (B)							
Treatments	Odds ratio (95% credible intervals)						
	CTL	TDN	ONN	VAR	BUP	CYT	EC
CTL		1.66 (1.02, 2.67)	2.38 (1.37, 4.21)	2.95 (1.87, 4.65)	1.86 (0.92, 3.71)	2.26 (1.17, 4.48)	2.24 (1.25, 4.01)
TDN	0.6 (0.37, 0.98)		1.45 (0.7, 3.04)	1.77 (1.08, 3.02)	1.12 (0.54, 2.4)	1.35 (0.65, 2.94)	1.35 (0.67, 2.64)
ONN	0.42 (0.24, 0.73)	0.69 (0.33, 1.43)		1.24 (0.61, 2.68)	0.78 (0.31, 1.94)	0.94 (0.4, 2.37)	0.94 (0.45, 1.86)
VAR	0.34 (0.22, 0.53)	0.56 (0.33, 0.92)	0.81 (0.37, 1.65)		0.63 (0.32, 1.28)	0.76 (0.42, 1.47)	0.76 (0.36, 1.54)
BUP	0.54 (0.27, 1.08)	0.89 (0.42, 1.85)	1.28 (0.51, 3.22)	1.59 (0.78, 3.15)		1.22 (0.5, 3.11)	1.2 (0.48, 2.88)
CYT	0.44 (0.22, 0.86)	0.74 (0.34, 1.55)	1.07 (0.42, 2.48)	1.32 (0.68, 2.41)	0.82 (0.32, 2)		0.99 (0.4, 2.37)
EC	0.45 (0.25, 0.8)	0.74 (0.38, 1.49)	1.06 (0.54, 2.24)	1.32 (0.65, 2.74)	0.84 (0.35, 2.06)	1.01 (0.42, 2.47)	
DIC	80.63						
I^**2**^	0						
Tau^**2**^	0.23						

Treatment drop-out rates (C)							
Treatments	Odds ratio (95% credible intervals)						
	CTL	TDN	ONN	VAR	BUP	CYT	EC
CTL		1.05 (0.81, 1.36)	0.77 (0.53, 1.11)	0.9 (0.71, 1.19)	0.91 (0.64, 1.3)	1.3 (0.82, 2.02)	0.7 (0.46, 1.07)
TDN	0.95 (0.74, 1.24)		0.74 (0.47, 1.16)	0.87 (0.64, 1.19)	0.87 (0.59, 1.31)	1.25 (0.76, 2.04)	0.67 (0.41, 1.08)
ONN	1.3 (0.9, 1.88)	1.36 (0.86, 2.15)		1.18 (0.76, 1.83)	1.19 (0.7, 2.03)	1.68 (0.94, 3.08)	0.91 (0.55, 1.59)
VAR	1.11 (0.84, 1.41)	1.15 (0.84, 1.56)	0.85 (0.55, 1.32)		1.01 (0.68, 1.47)	1.43 (0.91, 2.17)	0.77 (0.47, 1.24)
BUP	1.1 (0.77, 1.56)	1.15 (0.76, 1.71)	0.84 (0.49, 1.42)	0.99 (0.68, 1.46)		1.44 (0.83, 2.45)	0.77 (0.45, 1.35)
CYT	0.77 (0.5, 1.21)	0.8 (0.49, 1.32)	0.6 (0.32, 1.06)	0.7 (0.46, 1.1)	0.7 (0.41, 1.21)		0.54 (0.3, 0.97)
EC	1.42 (0.94, 2.17)	1.49 (0.93, 2.41)	1.1 (0.63, 1.83)	1.29 (0.81, 2.11)	1.29 (0.74, 2.22)	1.86 (1.03, 3.36)	
DIC	139.61						
I^2^	6%						
Tau^2^	0.11						

TDN, transdermal nicotine; ONN, oronasal nicotine; VAR, varenicline; BUP, bupropion; CYT, cytisine; CTL, controls; DIC, deviance information criterion.

#### 3.2.2 Continuous smoking abstinence (CSA)

Figures 2B, 3B described the network of ***CSA***, comprising 42 study cohorts and 18,609 participants. Using the same algorithm described before, the random effect model was selected for the final report due to significantly lower DIC (77.67 in random, 140.62 in fixed model). As presented in Table 2, all comparative interventions except for ***BUP*** were associated with significant efficacy for the outcome of ***CSA*** compared with ***CTL***. Similar to the absolute values of OR in analyses of ***PSA***, VAR (OR 3.02, 95% Crl 1.9–4.81) and ***TDN*** (OR 1.83, 95% Crl 1.09–3.17) demonstrated the highest and lowest OR, respectively. Tau^2^ in the analyses of ***CSA*** was estimated to be 0.24, indicating a moderate variance; and the I^2^ was estimated to be 0.00%, indicating that heterogeneity was minimally considerable. As presented in Figure 3B, inconsistency between direct and indirect evidence was observed in the comparison of VAR/***CTL***, ***CYT***/***CTL***, and ***VAR***/***CYT***. Among those inconsistent results, the direct evidence of ***VAR***/***CTL*** (OR 4.23, 95% Crl 2.57–6.77) yielded a positive ***CSA*** reduction on Varenicline use, but the direct evidence of ***CYT***/***CTL*** (OR 1.13, 95% Crl 0.508–2.52) and ***CYT***/***VAR*** (OR 1.13, 95% Crl 0.618–2.15) were ambiguous compared with their combined evidence. Comparative loops with e-cigarette (***EC/CTL***, ***EC*/*TDN***, and ***EC/ONN***) were exclusively consistent between direct and indirect evidence, and neither superiority nor inferiority was significant in ***EC/TDN*** (OR 1.25, 95% Crl 0.59–2.61) and ***EC/ONN*** (OR 0.96, 95% Crl 0.45–1.99) comparisons.

#### 3.2.3 Treatment drop-out rates (TDR)

Figures 2C, 3C described the network of ***CSA***, comprising 42 study cohorts and 18,609 participants. Following similar principles as before, we opted for a random-effects model as significantly lower DIC (80.63 in random, 140.62 in fixed model). As presented in Table 2, With the exception of BUP, all intervention groups exhibited significant superiority over the control group in terms of continuous abstinence rates. Among these, VAR, ***CYT***, ***EC***, and ***ONN*** showed odds approximately 2–3 times higher than the control group. Noneligible Tau^2^ in the analyses of ***CSA*** was estimated to be 0.24, indicating a moderate variance; and the I^2^ was estimated to be 0.00%, indicating that heterogeneity was minimally considerable. For the outcome measure of ***CSA***, comparisons among active intervention groups mirrored those of ***PSA***, with only VAR demonstrating significant superiority over ***TDN***.

### 3.3 Treatment ranking

As presented in Table 3, we estimated the posterior distribution of the ranking probability and their corresponding SUCRA for all outcomes. Briefly, ***CYT*** is quite likely to encourage both prevalent and continuous smoking abstinence but may lead to dropout. ***VAR*** is quite likely to encourage prevalent abstinence, is not particularly effective with continuous abstinence, and is very likely to prompt dropout. In contrast, ***ONN*** and ***EC*** are least likely to prompt dropout and both are more effective than no treatment in encouraging prevalent abstinence. However, ***TDN*** is more effective than no treatment in continuous abstinence, with neither significant effect on prevalent abstinence nor dropout rate (More details seen in Table 3).”

**Table 3:** Posterior distribution of the ranking probability and the surface under the cumulative rank curve (SUCRA) for each treatment in network meta-analysis.

Prevalent smoking abstinence (A)							
Rank	Treatment						
	BUP	CTL	CYT	EC	ONN	TDN	VAR
**1**	0.02	0.00	0.33	0.09	0.10	0.00	0.46
**2**	0.03	0.00	0.44	0.08	0.07	0.00	0.38
**3**	0.14	0.00	0.17	0.25	0.20	0.10	0.13
**4**	0.18	0.00	0.04	0.25	0.21	0.28	0.03
**5**	0.22	0.05	0.01	0.17	0.18	0.38	0.00
**6**	0.24	0.29	0.00	0.11	0.15	0.22	0.00
**7**	0.17	0.66	0.00	0.05	0.10	0.02	0.00
**SUCRA**	0.34	0.07	0.84	0.52	0.47	0.37	0.88

Continuous smoking abstinence (B)							
Rank	Treatment						
	BUP	CTL	CYT	EC	ONN	TDN	VAR
1	0.06	0.00	0.13	0.14	0.19	0.00	0.48
2	0.10	0.00	0.19	0.17	0.20	0.03	0.31
3	0.14	0.00	0.21	0.21	0.23	0.07	0.14
4	0.19	0.00	0.20	0.20	0.19	0.17	0.05
5	0.24	0.01	0.15	0.16	0.12	0.30	0.01
6	0.23	0.07	0.11	0.11	0.08	0.41	0.00
7	0.04	0.93	0.01	0.01	0.00	0.02	0.00
SUCRA	0.46	0.64	0.91	0.13	0.22	0.71	0.43

Treatment drop-out rates (C)							
Rank	Treatment						
	BUP	CTL	CYT	EC	ONN	TDN	VAR
1	0.05	0.14	0.14	0.18	0.24	0.17	0.08
2	0.04	0.23	0.37	0.26	0.10	0.01	0.00
3	0.76	0.11	0.06	0.04	0.02	0.01	0.00
4	0.00	0.02	0.03	0.04	0.09	0.25	0.57
5	0.01	0.03	0.04	0.08	0.15	0.38	0.31
6	0.13	0.40	0.22	0.14	0.09	0.02	0.01
7	0.01	0.07	0.14	0.27	0.31	0.16	0.04
SUCRA	0.46	0.64	0.91	0.13	0.22	0.71	0.43

TDN, transdermal nicotine; ONN, oronasal nicotine; VAR, varenicline; BUP, bupropion; CYT, cytisine; CTL, controls; SUCRA, surface under the cumulative rank curve.

## 4 Discussion

As far as we know, our study is the first to report the efficacy and acceptability of five major pharmacological monotherapies and e-cigarette on smoking cessation through network meta-analysis including RCT studies. And this NMA including 40 studies found that (1) Varenicline is more effective intervention to assist in smoking cessation during mid- to long-term (16–32 weeks) follow-up, but is not particularly effective with continuous abstinence, and is very likely to prompt dropout. (2) Cytisine shows more effectiveness in continuous smoking cessation but may lead to dropout. (3) E-cigarettes and oronasal nicotine are least likely to prompt dropout and both are more effective than no treatment in encouraging prevalent abstinence. Finally, transdermal nicotine delivery is more effective than no treatment in continuous abstinence, with neither significant effect on prevalent abstinence nor dropout rate.

Our findings are consistent with the approach recommended by current mainstream clinical smoking cessation guidelines, such as the use of Varenicline as a first-line pharmacological intervention to assist in smoking cessation by the 2020 American Thoracic Society guidelines (53) and the recommendation of NRT, varenicline and bupropion as first-line pharmacological interventions for smoking cessation in the 2018 ACC Expert Consensus (54). Since e-cigarettes (or equivalent products) have a pharmacological mechanism for distributing nicotine to the body, their potential cessation effect has also gained the attention of manufacturers. This study also showed their similar effects to NRT treatment in terms of smoking cessation effectiveness.

However, our findings should be cautiously interpreted. The ethnic distribution of overall participants involved in this NMA is considerably uneven since most of the included RCTs were performed in Europe and North America. Thus, the results of this NMA may not be generalized to other ethnical groups due to the differences in tobacco dependence and cessation in acculturation and nicotine metabolism levels described in previous studies (55, 56). Gender differences in pharmacotherapies of smoking cessation are also non-negligible since it is clear that certain medication shows different efficacy between male and female participants desiring smoking cessation (56). The selection criteria of smoking intensity and duration in each included study can vary considerably, ranging from light-intermittent to heavy-daily smoking, and such differences in smoking intensity may indirectly affect patients’ confidence in quitting smoking (57). A more specifically stratified discussion in participants with different smoking intensities should be investigated in further studies.

Overall, there is a moderate level of variance among all included studies. Such variance may result from several possible aspects. Firstly, interventions were artificially classified, and the oronasal nicotine replacement therapy includes four different FDA-approved pharmacotherapies (nicotine nasal spray, nicotine inhaler, nicotine gum, and nicotine lozenge) with possibly variant effectiveness due to different nicotine delivering dosages and delivering routes. Secondly, the overcall control group also has the potential of being part of the variance. We summarized all placebo groups and control groups without blind settings from each trial as one single group, and there may also be differences in the effect on treatment outcomes between the different placebo and the unblinded control settings. Additionally, it has been described by *Chan 2021,* etc. that the diversity of e-cigarette products may also be problematic to generalize the results to newly-created e-cigarette products (5). And last, of all, the method chosen for verification of outcome measurement is also concerning. Though serum and urine cotinine are used for smoking cessation verification and quantitative measurement in some trials, exhaled CO is the mostly applied biochemical method for the same purpose and has revealed several shortages, including short half-life (58), and false-positive results with other smoking products (e.g., cannabis) (59).

It has been proved by several previous meta-analysis and RCTs and has been validated by this NMA that e-cigarette is effective in assisting smoking cessation. Compared to ***PSA***, E-cig had a higher probability of superior ranking in smoking cessation effectiveness as measured by ***CSA***, and this similar finding is also observed in the oronasal nicotine group. Based on this finding, we hypothesized that patients who use e-cigarettes and oronasal nicotine products would have higher adherence due to their similar nicotine delivery pattern to conventional tobacco cigarettes. This hypothesis is also validated by the NMA of treatment drop-out rates which demonstrates the highest acceptance of e-cigarettes and oronasal nicotine treatment among all interventions.

However, we should still be cautious to approve e-cigarettes as a therapeutical intervention for smoking cessation. E-cigarette or vaping product use-associated lung injury (EVALI), a novel entity including a broad spectrum of pulmonary diseases and may lead to respiratory failure, has continuously been reported (60–66). There is also growing evidence indicating generalized pulmonary toxicity may be caused by inhaling electronic cigarette vapor (67). Additionally, the psychoactive substances and special flavors of vapor have led to a surge in usage, especially among adolescents (68). A study from the U.S. indicates that more than 40% of high school students have tried e-cigarettes in the past year in 2020 (69). What is more alarming is that studies have proved that initial e-cigarette use is also associated with subsequent cigarette smoking initiation among adolescents and young adults (70, 71). Further discussion on whether e-cigarettes can be used as a pros-outweigh-cons intervention in assisting smoking cessation should follow more investigations on their long-term safety. On balance, we have reservations about e-cigarettes as a way to quit smoking.

## 5 Conclusion

Our study reported the efficacy and acceptability of five major pharmacological monotherapies and e-cigarette on smoking cessation through network meta-analysis including 40 RCT studies. We recommended that Varenicline, Cytisine and transdermal nicotine delivery, as smoking cessation intervention, have advantages and disadvantages. However, we had to have reservations about e-cigarettes as a way to quit smoking in adolescents.

## Author contributions

YM: Writing – original draft, Writing – review & editing. SX: Writing – review & editing. LQ: Writing – original draft. YL: Writing – review & editing.
